# Ethical and Scientific Issues of Nanotechnology in the Workplace

**DOI:** 10.1289/ehp.9456

**Published:** 2006-09-25

**Authors:** Paul A. Schulte, Fabio Salamanca-Buentello

**Affiliations:** 1 National Institute for Occupational Safety and Health, Centers for Disease Control and Prevention, Cincinnati, Ohio, USA; 2 University of Toronto Joint Centre for Bioethics and Canadian Program on Genomics and Global Health, Toronto, Ontario, Canada

**Keywords:** ethics, hazards, nanotechnology, occupational safety and health, particles, toxicology

## Abstract

In the absence of scientific clarity about the potential health effects of occupational exposure to nanoparticles, a need exists for guidance in decisionmaking about hazards, risks, and controls. An identification of the ethical issues involved may be useful to decision makers, particularly employers, workers, investors, and health authorities. Because the goal of occupational safety and health is the prevention of disease in workers, the situations that have ethical implications that most affect workers have been identified. These situations include the *a*) identification and communication of hazards and risks by scientists, authorities, and employers; *b*) workers’ acceptance of risk; *c*) selection and implementation of controls; *d*) establishment of medical screening programs; and *e*) investment in toxicologic and control research. The ethical issues involve the unbiased determination of hazards and risks, nonmaleficence (doing no harm), autonomy, justice, privacy, and promoting respect for persons. As the ethical issues are identified and explored, options for decision makers can be developed. Additionally, societal deliberations about workplace risks of nanotechnologies may be enhanced by special emphasis on small businesses and adoption of a global perspective.

Science and technology have identified unique properties in materials with dimensions in the range of 1–100 nm [Health and Safety Executive (HSE) 2004; National Nanotechnology Initiative (NNI) 2004]. These properties may yield many far-reaching societal benefits, but they may also pose hazards and risks. One area of concern about hazards is the workplace—be it a research laboratory, start-up company, production facility, or operation in which engineered nanomaterials are processed, used, disposed, or recycled. These are the workplaces in which some of the first societal exposures to engineered nanoparticles are occurring. Such exposures are likely to be inadvertent and unintended. Despite a conscious effort by governments, corporations, nongovernmental organizations (NGOs), trade associations, academics, and workers to anticipate and address potential workplace hazards [Bartis and Landree 2006; Hett 2004; National Institute for Occupational Safety and Heath (NIOSH) 2006; National Science and Technology Council (NSTC) 2006; Roco and Bainbridge 2003; Scientific Committee on Engineering and Newly Identified Health Risks (SCENIHR) 2005], workers are still likely to be exposed to nanomaterials.

Much research on the ethical aspects of nanotechnology has focused on generalized issues such as equity, privacy, security, environmental impact, and metaphysical applications concerning human–machine interactions (Mnyusiwalla et al. 2003; Moor and Weckert 2004; Singer 2004). No ethics research has been carried out that pertains specifically to the workplace. To help anticipate the impact of nanotechnology, it is important to provide a framework for the ethical and scientific issues involved with nanotechnology in the workplace. Ethical analysis may assure society that the expansive promise of nanotechnology does not conceal hazards and risks for workers. An emerging belief is that nanoscience and technology cannot be based on past practices in which ethical and social reflection is a second step to using newly developed science; rather, ethical reflections must accompany research every step of the way (National Academy of Engineering 2004). Our goal in this paper is to identify ethical issues that are directly related to nanotechnology in the workplace and their implications for workers’ health and safety.

## Framework for Ethical Assessment

The framework for considering the ethical issues can be drawn from the work of Gert et al. (1997), Gewirth (1978, 1986), and Schrader-Frechette (1994) as well as from the “principlist” approach of Beauchamp and Childress (1994). The ethical issues that most affect workers in jobs involving nanomaterials are linked to identification and communication of hazards and risks by scientists, authorities, and employers; acceptance of risk by workers; implementation of controls; choice of participation in medical screening; and adequate investment in toxicologic and exposure control research (Table 1). The ethical issues involve the identification and assessment of hazards and risks, nonmaleficence (doing no harm), autonomy (self-determination), justice (fairness in distribution of risks), privacy (in handling of medical information), and respect for persons.

Factual scientific knowledge—which is the basis for ethical decisions about occupational safety and health—may be influenced by biases and values (Kantrowitz 1995). Scientific knowledge is unavoidably value laden. No scientific theory can be considered to be wholly objective, but one theory may be more objective than another (Shrader-Frechette 1994). Underlying the ethical decisions are the way in which nanotechnology is depicted, the potential benefits, and the associated hazards and risks. When information about the hazards of nanoparticles is in doubt, the critical question is where to draw the line about the necessary level of protection and the residual risk at a given level of protection.

Risk assessments are partly subjective and likely to be highly politicized. Thus all risk projections are value laden. No single scenario for describing risks and controls can suffice because of the heterogeneous and developmental nature of nanotechnology. The ethical issues will be specific only for the knowledge base at a given time and for a specified production and use scenario. Researchers have suggested that even with that type of specificity, alternative assessments are needed to capture the ethical and political values that inform policies such as those involving nanotechnology (Schrader-Frechette 2002).

## Current State of Knowledge about Nanotechnology Hazards and Risks

The way in which nanotechnology is depicted may influence society’s reactions to research, development, and prevention and control of potential nanomaterial hazards in the work-place (Berube 2004). The term “nanotechnology” is misleading, since it is not a single technology but a multidisciplinary grouping of physical, chemical, biological, engineering, and electronic processes, materials, applications, and concepts in which size is the defining characteristic (Aitken et al. 2004). However, the issues of size, surface characteristics, durability, chemical composition, and other physiochemical features are not well resolved in the definition. A fuller definition also includes structures with novel properties that can be manipulated on the atomic scale (NNI 2004; Salamanca-Buentello et al. 2005).

Nanoparticles can be considered in at least two broad categories: engineered nanoparticles and incidental (or adventitious) nanoparticles. Engineered nanoparticles are designed with very specific properties. Incidental nanoparticles (natural and anthropogenic) are generated in a relatively uncontrolled manner and are usually physically and chemically heterogeneous compared with engineered nanoparticles (NIOSH 2006). Although the four current major production methods of engineered nanoparticles (gas-phase synthesis, vapor deposition, and colloidal and attrition methods) may expose workers by inhalation, dermal absorption, and ingestion, the amount and likelihood of worker exposure has not been well established. The critical question (based on the little information available) pertains to the assessment of hazards and risks. The unifying theme is that nanoparticles are smaller than their bulk counterparts but have a larger surface area and particle number per unit mass; these characteristics generally increase toxic potential as a result of increased potential for reactivity (Aitken et al. 2004). The application of that theory to the whole of nanotechnology rather than to specific particles and processes may increase rather than decrease the uncertainty about hazards and risks. Increasingly, other characteristics (e.g., surface characteristics) in addition to particle size, that influence toxicity are being identified (Donaldson et al. 2006; Warheit et al. 2004). These characteristics are tremendously variable. Consequently, it is useful to put some limits on the uncertainty by being more precise in the language used to describe nanoparticle hazards and risks. Because a diverse mix of particles and processes exists, hazards and risks are likely to be more accurately assessed on a case-by-case basis—or at least according to the type of production methods and whether particles are embedded in a matrix or unbound.

### Knowledge about hazards and risks

Health effects data on workers involved with nanotechnology are limited because of the incipient nature of the field, the relatively small number of workers potentially exposed to date, and the lack of time for chronic disease to develop and be detected. The most relevant human experience deals with exposures to ultrafine particles (which include particles with diameters < 100 nm) and fine particles (particles with diameters < 2.5 μm). Ultrafine and fine particles have been assessed in epidemiologic air pollution studies and in studies of occupational cohorts exposed to mineral dusts, fibers, welding fumes, combustion products, and poorly soluble, low-toxicity particulates such as titanium dioxide and carbon black (Maynard and Kuempel 2005; Nel et al. 2006). The hazards of these exposures and exposures to engineered nanoparticles are also identified in animal studies (Donaldson et al. 2004, 2006; Elder et al. 2006; Lam et al. 2004, 2006; Oberdörster et al. 2005; Shvedova et al. 2005; Warheit et al. 2004). A strong relationship exists between the surface area, oxidative stress, and proinflammatory effects of nanoparticles in the lung. The greater the oxidative stress, the more likely the risk of inflammation and cytotoxicity (Nel et al. 2006; Oberdörster et al. 2005). The findings from animal studies ultimately need to be interpreted in terms of the exposure (dose) that humans might receive. Although there is still some debate, the evidence from air pollution studies associates increased particulate air pollution (the finer particulate matter fraction, PM_2.5_, with an aerodynamic diameter < 2.5 μm) with adverse health effects in susceptible members of the population—particularly the elderly with respiratory and cardiovascular diseases [Mark 2004; Peters 2005; U.S. Environmental Protection Agency (U.S. EPA) 2004]. Moreover, the concentrations associated with measurable effects on the health of populations are quite low (Aitken et al. 2004).

In occupational studies, the populations that are repeatedly exposed to hazardous mineral dusts and fibers in the respirable range (e.g., quartz and asbestos, respectively) have well-known health effects related to the dose inhaled (Maynard and Kuempel 2005). With asbestos, the critical risk factors for developing respiratory diseases are fiber length, diameter, and biopersistence. For poorly soluble, low-toxicity dusts such as titanium dioxide, smaller particles in the nanometer size range appear to cause an increase in risk for lung cancer in animals on the basis of particle size and surface area (Heinrich et al. 1995; Oberdörster et al. 2005; Tran et al. 2000).

Although the findings are not conclusive, various studies of engineered nanoparticles in animals raise concerns about the existence and severity of hazards posed to exposed workers (Kipen and Laskin 2005). Possible adverse effects include the development of fibrosis and other pulmonary effects after short-term exposure to carbon nanotubes (Lam et al. 2006; Oberdörster et al. 2005; Shvedova et al. 2005), the translocation of nanoparticles to the brain via the olfactory nerve, the ability of nanoparticles to translocate into the circulation, and the potential for nanoparticles to activate platelets and enhance vascular thrombosis (Radomski et al. 2005).

None of these findings are conclusive about the nature and extent of the hazards, but they may be sufficient to support precautionary action.

Ultimately, the significance of hazard information depends on the extent to which workers are exposed to the hazard. This is the defining criterion of risk (the probability that an exposed worker will become ill). A need has been identified for nanoparticle-specific risk assessments (i.e., those that use the most appropriate dose metrics rather than typical mass) that will be unique to nanotechnology (National Academy of Engineering 2004; SCENIHR 2005).

Risk assessment has been widely used to manage the uncertainty of risks posed to humans by newly introduced chemicals or processes. However, nanotechnology encompasses a diverse range of compositions, structures, and applications, so a single risk assessment and management strategy may not be appropriate (Wardak and Rejeski 2003). Nanotechnology involves the manipulation of matter at the nanoscale to produce materials, structures, and devices that contain various particle types, sizes, surface characteristics, and coatings. These particles may best be addressed by a range of risk assessments specific to the type of particle (composition, surface characteristics, and shape) being assessed. Because of the general inverse relationship between particle size and surface area, dose–effect relationships may vary as a function of total surface area and number of particles rather than mass units (SCENIHR 2005). Risk assessments will be useful to the extent that they reflect the effects of particle sizes and surface area, but such assessments may also need to reflect other particle characteristics. Moreover, it is currently unclear the extent to which the toxico-kinetics (an important component in risk assessment) can be predicted from knowledge of physicochemical properties of nanoparticles (SCENIHR 2005).

### Evidence base for hazard controls

The most frequently used model of the workplace environment identifies sources of hazards and routes of exposure (e.g., inhalation, skin) [Office of Technology Assessment (OTA) 1985]. Control can be introduced at each of these points. Occupational safety and health professionals have identified a hierarchy of controls based on reliability, efficiency, and the principle that the environment should be controlled before the worker is required to take any preventive action (OTA 1985). In its simplest form, the hierarchy of controls specifies that engineering controls (including substitution, enclosure, isolation, and ventilation) are preferred to the use of personal protective equipment (such as protective clothing and respirators). Work practices are frequently incorporated in risk management efforts to minimize worker exposures, and they often supplement the use of engineering controls. Administrative controls such as worker rotation are sometimes included and generally constitute the “third line of defense” when engineering controls and work practice controls cannot achieve the desired level of worker protection (OTA 1985).

**Figure 1:**
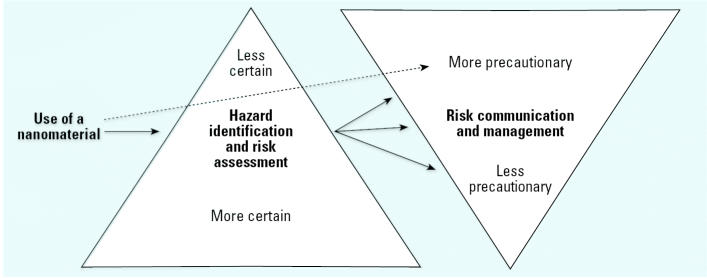
Risk management decisionmaking for nanoparticles in the workplace: what is the appropriate level of controls?

In the absence of adequate toxicity information and extensive history of engineered nanomaterials use, the rationale for control guidance has been based on experience in controlling exposures to incidental ultrafine particles and gases. Airborne nanoparticles are considered to have no inertia—hence, they will behave similarly to gases and will diffuse if they are not fully enclosed (Aitken et al. 2004). A rich history of aerosol science describes the fundamental properties of aerosols and their control [American Conference of Governmental Industrial Hygienists (ACGIH) 2001; Brown 1993; Burton 1997; Davies 1966; Friedlander 1977; Fuchs 1964; Hinds 1999; Ratherman 1996]. Although ultrafine particles are considered equivalent to nanoparticles by some authorities (SCENIHR 2005), they are usually (but not exclusively) at the upper end of the nanoscale range. If airborne nanoparticles conform to the classical physics and aerodynamics observed for larger particles, then controls effective in capturing fine and ultrafine particles and gases (such as source enclosure, local exhaust ventilation, and personal protective equipment) should be effective with the current generation of nanomaterials. It is reasonable to believe that most control methods used for fine and ultra-fine particles and also for gases will be useful for controlling nanoparticles, but there is no reason to expect that application of these methods to new nanoparticle generation processes will result in better control than that previously demonstrated for microscale powders and gases (Aitken 2004). A considerable body of opinion indicates that the adverse effects of nanoparticles cannot be predicted (or derived) from the known toxicity of bulk materials with similar chemical composition and surface properties (SCENIHR 2005). Control options for nanoparticles range from no controls to the use of isolation and containments practiced with radiation, gases, and biological agents. The question is where in this continuum should controls be selected. This may also translate into how much money to invest in them. When risks are known to be high or low, the decision is relatively easy, and the appropriate control strategies are generally apparent. However, when hazards are uncertain (as they are with nanoparticles), the difficulty is in deciding what level of controls is warranted (Figure 1). Given the paucity of toxicity information, control guidance must be regarded as interim, and some authorities believe that it should be precautionary—that is, tending toward reducing exposures as much as possible (HSE 2004).

### Summary of evidence on hazards and controls

The evidence base pertaining to nanotechnology hazards and controls has been reviewed in various publications (Hett 2004; Maynard and Kuempel 2005; National Academy of Engineering 2004; NIOSH, 2006; Royal Society and Royal Academy of Engineering 2004; SCENIHR 2005) and is summarized in Table 2 by four categories of knowledge described in terms of hazards and controls and awareness. These categories are mutable and pertain to the state of knowledge at a given time. Category 1 (“what we know we know”) indicates that we have some knowledge about the health hazards posed by some types of nanoparticles (e.g., ultrafine particles) and gases and how to control them. This category applies to the current generation of engineered nanoparticles and is the basis for much of the current guidance. Category 2 knowledge (“what we know we don’t know”) is the basis for much of the research currently being conducted or planned. In general, we do not know much about the hazards of new or anticipated engineered particles or whether enough precautions have been taken. A major question is not only how to control exposure but also what are the appropriate extent and cost of controls. Category 3 knowledge (“what we don’t know we know”) represents the under-utilization of established knowledge. That is, scientists have had extensive experience in hazard and exposure control for ionizing radiation, biological agents, pharmaceuticals, grain and mineral dusts, and air pollution. This experience could be more directly brought to bear on controlling the hazards of nanomaterials in the workplace. In addition, this category could include proprietary information about nanoparticles that is not available for hazard assessments. Category 4 knowledge (“what we don’t know we don’t know”) represents a perennial area of philosophical exploration (Caws 1998). This category includes the range of scenarios about the potency of hazards and the extent of risks. Will new scenarios present new types of exposures and risks? The popular literature on nanotechnology is replete with characterizations of possible future scenarios, but no projections have been made of workplace hazards and risks (Drexler 1986; Regis 1995). Category 4 knowledge also includes the lack of awareness of factors influencing an issue. This lack of awareness can be addressed by engaging a wide variety of disciplines and communities of interest to characterize an issue (HSE 2004). Category 4 knowledge also includes the beliefs we hold that may be wrong. Such beliefs could lead to taking or not taking protective measures on the basis of faulty assumptions. Eventually, Category 4 knowledge can be transformed to Category 2 and then to Category 1.

Regardless of which type of knowledge is considered, the ultimate ethical requirement is to accurately portray the state of knowledge about a hazard or risk and not to understate or overstate it. However, given the developmental nature of nanotechnology, the knowledge of hazard potential will change over time and require restatement and possibly modification of guidance. In the absence of adequate hazard and risk assessment data, the critical question is how much caution is warranted.

## Ethical Issues

### Identifying and communicating hazards and risks

The “hazard identification” stage of risk analysis is the basis for risk management decisionmaking. The output of this stage is often highly debated, since the process of reasoning is primarily qualitative and the results trigger other stages of analysis and decisions about preventive action (Crawford-Brown and Brown 1997). Interpreting scientific information about the hazards of nanomaterials is basic to communicating the hazards and risks posed to workers. Interpreting and communicating hazard and risk information is an integral part of risk management by employers. The employers’ decisionmaking will focus on deciding which preventive controls should be used to assure a safe and healthful workplace.

Employers and workers look to scientists and authoritative organizations to help interpret hazard and risk information and to put it into context. This expectation may pressure scientists to go beyond the mere conduct of research. The interface between science and morality is exceedingly complex, but scientists are generally considered to have ethical obligations to society at large (Pimple 2002; Schrader-Frechette 1994; Weil 2002). However, no consensus has been reached about the nature of those ethical obligations beyond fulfilling the professional responsibilities internal to scientific research. Framing a clear and coherent approach to the ethical responsibilities of scientists in nanotechnology is a difficult task. At the least, such an approach requires scientists to use appropriate qualifiers in published papers and to be cautious in generalizing their results. More broadly, it means not shrinking from considering the implications of their work, even if all the scientific details are not known.

Decision makers may have inadequate scientific information to help them decide how precautionary their approach should be (Cairns 2003). To determine whether a decision conforms with the principle of nonmaleficence, decision makers must determine the harm that could occur if the nanoparticles were as toxic as suggested by preliminary hazard information. Data on air pollution and industrial ultrafine particles indicate that a given mass of nanoparticles would be more biologically reactive and hence potentially more toxic than the same mass of larger particles (Seaton 2006). Consequently, the level of control might need to be more stringent for smaller nanoscale dusts than for those with diameters > 100 nm. Ultimately, the more stringent level of controls may result in risks that are equal to or smaller than risks posed by larger particles. Authoritative organizations and employers are responsible for communicating the risk workers face after appropriate controls are implemented. Failure to do so may preclude workers from exercising autonomy. This issue may be confounded by the fact that the employer has a proprietary interest in not releasing information about “nanoproducts” and workplace controls.

The principlist ethical approach focuses on principles such as nonmaleficence and autonomy but fails to assess the social and organizational context of occupational safety and health and the role of practitioners in relation to the corporate structure (Gert et al. 1997; Samuels 2003). With regard to nanotechnology, the contextual pressures on practitioners and authorities arise from a company’s or society’s needs and desires for nanotechnology to grow and develop. Mention of potential health concerns may be seen as alarmist, unfounded, and detrimental to the growth of the field. Nonetheless, the counter position is that conflicting demands on practitioners from being both an agent of a company and an autonomous professional constitute a social and structural problem rather than a problem of individual ethics (Draper 2003; Samuels 2003). One solution is that health pronouncements be made independently of promotional concerns for nanotechnology.

### Workers’ acceptance of risk

Acceptance of risk is a relative concept that includes judgment about the certainty and severity of risk, the extent of the health effects, voluntary nature of the risk, the risks and advantages of any alternatives, and compensation for undergoing the risk (Fischoff 1994). It is a false premise to assert that workers have free choice in terms of which work and working conditions to accept. Although some component of self-determination is present, economic and social conditions exert the greatest influences on workers’ selection of work, level of risk tolerated, and ability to participate in risk management. Worker participation in risk management is not a static concept and has increased over the past 35 years with the implementation of team approaches, management systems, corporate responsibility, and right to know and act movements (Gallagher 1997; Jensen 2002; Lynn 1997; Shearn 2005). Nonetheless, workers generally cannot universally refuse work they consider hazardous and still keep their jobs. Conformance with the principle of autonomy depends on the extent to which workers have input into risk management at their work sites and the degree to which they are at risk after controls have been implemented.

Justice is also related to worker decision-making. At issue is the extent to which workers are exposed to greater risks than the general public—or, stated another way, whether it is appropriate to exchange incentives such as wages or hazardous duty pay for additional risk from exposure to nanoparticles (Schrader-Frechette 2002). This issue may be less significant if nanoparticle controls reduce workers’ risk levels to those of the general public, if conceivably both are known. Clearly, society accepts that some jobs are inherently riskier than others. However, in many countries the societal goal is to provide a safe and healthful workplace for all workers.

### Selecting and implementing controls

The critical ethical question related to control of nanoparticles is whether sufficient controls are being implemented to prevent injury and illness. If not, worker exposures may result in increased risk of harm or actual harm. The central scientific fact is that the risk posed by nanomaterials is not well established. However, preliminary information suggests that at least the same level of concern afforded to industrial fine and ultrafine particles should be extended to engineered nanomaterials and that a commensurate level of protection should be instituted for them (Hett 2004; Royal Society and Royal Academy of Engineering 2004; Seaton 2006). Any risk posed by exposure to ultrafine particles is a function of their potential toxicity and the extent of exposure. Based on limited toxicological evidence of risk and a heightened level of concern, the best approach might be to treat engineered nanoparticles as if they were potential occupational hazards and to use a prudent health-protective, risk-based approach to develop interim precautionary measures consistent with good professional occupational safety and health practice (Royal Society and Royal Academy of Engineering 2004).

Such interim precautionary measures could include guidelines for conducting work-place exposure assessments, implementing engineering controls, designating work practices, and developing process or industry interim exposure limits as core elements. If the focus of exposure control is airborne particles of respirable dimensions, such approaches may be useful and reflect the professional judgment of experienced practitioners. If skin absorption is also a likely route of exposure, guidelines should be developed for preventing skin exposure. Unfortunately, data are insufficient to make a strong risk-based assessment to inform these decisions.

The evidence suggests that at least some manufactured nanoparticles will be more toxic per unit of mass than larger particles of the same chemicals (Royal Society and Royal Academy of Engineering 2004). However, some evidence indicates that with the use of existing controls for fine or ultrafine particles, workers will not be at inordinately elevated risk for lung disease. For example, estimates based on animal studies indicate that workers exposed to ultrafine titanium dioxide at 0.1 mg/m^3^ for a 45-year working lifetime have an excess risk of lung cancer that is < 1/1,000 and could in fact have a risk approaching zero (Kuempel et al. 2004). The basis for these findings is the hazard posed by increased particle surface area for a given mass of small-sized particles, as derived from animal studies and extrapolated to humans. The extent to which this analysis pertains to other nanoparticles is not known and may vary depending on morphology, surface activity, and biopersistence. Moreover, precise risks from exposure to these ultrafine particles can be determined only if adequate animal or human data are available. Also, if particles can translocate into the central nervous system or the circulatory system, further estimates will be required before conclusions can be drawn (Oberdörster et al. 2005).

In short, given the insufficient evidence of hazards posed by the current generation of nanoparticles, the risks (whatever they may be) are expected to be reduced when controls recommended for known industrial ultrafine particles (such as titanium dioxide) are utilized. This conclusion is supported by *a*) a generalized risk assessment based on surface area for poorly soluble, low-toxicity particles and *b*) the fact that such particles conform to classic physics and aerodynamic laws when airborne. However, future assessments of risk could be different, depending on the bio-persistence, structure, surface activity of new particles, and information about translocation across endothelial cell barriers. If these topics are the focus of risk communications and management efforts, there appears to be general conformance with the ethical principles of beneficence and nonmaleficence. At the same time, no strong evidence indicates that workers in these environments are not at excess risk. Minimal risk is only assumed on the basis of qualitative risk assessments and the utility of proven controls for some types of particles.

Overall, the knowledge base pertaining to nanomaterials is not static but changes as scientists develop new materials and conduct toxicological or other health effects research. Consequently, ongoing evaluation of health risks is needed along with continued communication and development of management plans to be in conformance with the ethical principles discussed in this article.

### Establishing medical screening programs

Medical screening is the application of tests to asymptomatic persons to detect those in the early stages of disease or at risk of disease. Medical screening in the workplace differs from medical screening in the general population because of the specific nature of the occupational condition and responsibilities of employers (Halperin et al. 1986; Harber et al. 2003). A wide range of ethical questions has been identified regarding the medical screening of workers and the use and implications of the findings (Ashford et al. 1990; Schulte 1986). These questions address the rationale for screening, the voluntary nature of the screening, the action that will be taken for workers with positive tests, and individuals who will have access to test information.

Medical screening is not generally warranted when the toxicity of a material and the workers’ risk are unknown—as is the case with most nanomaterials. Moreover, for diseases such as lung cancer (which is a potential outcome resulting from some nanoparticle exposure), no strong evidence base exists for routine screening; and general population screening for lung cancer is not generally recommended [National Cancer Institute (NCI) 2006]. Not only does screening fail to reduce mortality from lung cancer, it could lead to false-positive tests and unnecessary invasive procedures or treatments (NCI 2006). Medical screening of workers may be warranted for nonmalignant respiratory effects in some nanotechnology operations where significant residual risks may occur after controls are implemented. Such screening should be part of a comprehensive risk management program that considers not only respiratory hazards but also cardiovascular and neurologic risks as well as risks in various other potential target organ systems (Oberdörster et al. 2005; Radomski et al. 2005; Tran et al. 2005). If various nanomaterials are found to have toxic effects and if appropriate (validated) tests exist for early detection of those effects in exposed workers, medical screening might be warranted. However, medical screening is historically viewed as a secondary preventive effort in the hierarchy of controls (Ashford et al. 1990)

The ethical questions that apply to the medical screening of workers pertain to whether the screening is voluntary, who will have access to the results, and what the purpose of such access will be. Screening generally requires diagnostic confirmation; and for positive cases, screening requires timely treatment. Who is financially responsible for these procedures? Ethical issues can also arise in the use of screening results to label or stigmatize workers or to remove them from a job. Screening results may also create psychological burdens. Resolving such ethical issues will depend partly on the degree to which the worker has been informed about how the results will be used.

### Ensuring adequate investment in toxicological and control research

Ethical issues cannot be adequately addressed for nanotechnology without sufficient knowledge of the hazards involved. Because limited information is available on the safety of an ever-growing number of nanomaterials, an ongoing research effort is needed to comport with the principles of autonomy, beneficence, and nonmaleficence. In addition, research is needed on the extent of exposure and the effectiveness of controls. Internationally, such research is under way.

However, the question of the level of funding of this research has ethical implications because much of the current control guidance is precautionary and is not based on strong quantitative risk assessments. Further research is the only way to address this lack of appropriate information.

Some commentators have called for a slowdown in research and development of nanoparticles, whereas others have identified a need for increased health effects research and ethical analysis [Action Group on Erosion, Technology and Concentration (ETC Group) 2003, 2004; Mnyusiwalla et al. 2003]. The needs for health-based research have been identified and include the following topics: exposure and dose, toxicity, metrology, epidemiology, control technology, safety, education, recommendations, and applications in the near term (NIOSH 2006).

Researchers could help further the discussions of ethical issues by assessing the global budget for nanotechnology research and development and by determining the actual amounts dedicated to occupational safety and health research and ethical research in this field. Globally, such information is not well documented; but existing U.S. data can be considered. For the first time since the inception of the NNI, funding for 2005 was classified by program component area. The funding for the Societal Dimensions component area included $US39 million for environment, health, and safety and $43 million for educating the public about the broad implications of nanotechnology for society (including economic, workplace, education, ethical, and legal implications). This funding came from 11 agencies with a combined nanotechnology budget of approximately $1.054 billion. The level of funding (7.8% of the total) has been criticized as insufficient for the societal dimensions component and the subset dedicated to occupational safety and health (Bartis and Landree 2006; Maynard 2006; Service 2005). Nonetheless, there is a concerted international effort to address health and safety aspects of nanomaterials (NSTC 2006; Thomas et al. 2006).

### Promoting respect for persons

Underlying the debates about nanotechnology has been the issue of tolerating the potential for harm to some in the context of anticipated benefits to society. Such thinking embodies the utilitarian point of view that harm to one person may be justified by a larger benefit to someone else (Harris 2003). This point of view contrasts with the ethical principle of respect for persons, which emphasizes the rights of the individual and is associated with the golden rule (“Do unto others as you would have them do unto you”) (Gewirth 1978, 1986). In the workplace, this principle translates to acknowledging for each worker the right to a safe and healthful work environment. This right imposes correlative duties on the employers and governments who must secure the workers’ rights to a safe and healthful workplace (Gewirth 1986). The objection to this interpretation is that the rights of employers, and hence the rights of society, to property and benefit resulting from nanotechnology may be (or may appear to be) in conflict with workers’ rights. When two rights conflict with each other, some rational way must be found to determine their relative priority. Gewirth (1986) identified an essential criterion for such priority as the degrees of necessity for action. For example, where the property rights of employers may be in conflict with workers’ rights to safety and health, the diminution of health or a threat to safety lowers one’s capacity for action and is a greater loss than some decrease in another’s property, wealth, or freedom to control it. The practical implication is this: In the absence of adequate information about nanotechnology hazards, risks, and controls, employers should be moved to use more rather than fewer control measures (Hett 2004). Conducting site-specific hazard assessments and using appropriate controls appear to demonstrate conformance with the principle of respect for persons and with the principles of autonomy, beneficence, and nonmaleficence. However, the extent of control measures required may be the key matter of dispute. For the most part, control of the current generation of most engineered nanoparticles is within the capabilities of existing technologies. The issue is how much to invest in applying those technologies in a given workplace.

## Strategies for Supporting Ethical Decisionmaking

### Placing special emphasis on small businesses

The occupational safety and health problems of small businesses have been a major focus of concern, particularly in the last decade, since most workplaces are classified as small (i.e., workplaces that employ fewer than 250, 100, or 20 workers, depending on the definition). This statement is likely to hold true for work-places involving nanotechnology, but it is not well documented (Aitken et al. 2004; Roco and Bainbridge 2003). The frequency of occupational injury and illness in small businesses may exceed the average for general industry across all businesses in a sector, but the frequency may not be evident in an individual company (NIOSH 1999). Small businesses are generally perceived to have little time and few resources dedicated to occupational safety and health.

Small businesses are the driving force of most economies, including the subset of economies related to nanotechnology (Roco and Bainbridge 2003). Independent consultants, trade associations, insurance companies, product suppliers, and government agencies are the major sources of occupational safety and health information for small businesses. Occupational safety and health information may also be passed to downstream users of nanoparticles from upstream suppliers. In fact, for documented hazards, suppliers may have an ethical or legal obligation to pass on such information to downstream customers. There is a need for occupational safety and health guidance information about nanotechnology hazards and controls for small businesses.

### Adopting a global perspective

The growth of nanotechnology is a global phenomenon that requires a global approach to hazards and risks, particularly in the workplace. The world needs internationally valid standards for nanotechnology materials as well as a uniform nomenclature (American Society for Testing and Materials 2005; Hett 2004). Without a uniform nomenclature, investigators, insurers, regulators, governments, companies, and workers could have difficulty communicating and taking concerted actions.

The flow of materials in the global economy crosses many borders, including those of developing nations (Salamanca-Buentello et al. 2005). Thus, to assure the safety and health of workers, decision makers (whether they are employers or government authorities) must know and understand what materials are used in various processes and operations. This issue is complicated because many different definitions and descriptions may be used in science-based and regulation-based documents. To develop nanotechnology with minimal risks, knowledge gaps must be identified and addressed through international cooperation. Also needed is a transparent risk assessment framework that can achieve wide acceptability (SCENIHR 2005).

Global approaches to sharing occupational safety and health information require increased opportunity and capacity to access information. The “right” to know about risks—or more broadly, the right to information—is not evenly recognized worldwide (Pantry 2002). The World Health Organization (WHO) promotes the right to health at work for all. Information is a means to realizing that right. Despite broad WHO membership by many countries, true access to information and distribution within countries is still a problem.

Risk communications (including material safety data sheets) should reflect a degree of uniformity worldwide. International collaboration is warranted to ensure that hazardous processes are not relegated to countries with cheap labor markets or lax environmental controls (Singer et al. 2005, 2006). A critical issue that has both national and global implications is whether countries will treat nanomaterials made of a given substance differently from materials made with larger particles of the same substance. The characteristics of nanoparticles may be different from those of the larger particles with the same composition. For example, most materials made from carbon generally appear to pose a minimal health risk; however, nanotubes made of carbon may pose a greater health risk yet be regulated at the less protective level (Shvedova et al. 2005). The issue is whether to recommend the same risk communication and management strategy for both. On the basis of the carbon nanotube example, new standards and risk communication materials are likely to be required for at least some nanoparticles.

## Conclusions

The ethical questions about nanotechnology in the workplace arise from the state of knowledge about the hazards of nanomaterials and the risks they may pose to workers. The lack of clarity on these issues requires an interim assessment of the hazards and risks that might exist in various situations. Workers will be able to exercise their autonomy only if the processes leading to hazard identification and risk assessment are transparent and understandable. Employers will conform to the principles of autonomy, beneficence, nonmaleficence, justice, privacy and respect for persons to the extent that they *a*) accurately portray hazards and risks, *b*) are precautionary in their approach to hazards, *c*) engage in communication and dialogue with workers, and *d*) take the necessary steps to control risks so that they appear reasonable and acceptable to workers.

## Figures and Tables

**Table 1:** Ethical issues pertaining to workplace situations involving nanomaterials.

Work-related scenarios	Ethical principles involved	Decisionmaking issues
Identification and communication of hazards and risks	Responsibilities of scientists Nonmaleficence Autonomy Respect for persons	Extent to which strengths and weaknesses of data are identified Degree of participation in public discussion Accuracy of communications Timeliness of communications
Workers’ acceptance of risks	Autonomy Respect for persons Justice	Extent of inclusion of workers in decisionmaking
Selection and implementation of workplace controls	Nonmaleficence Beneficence Respect for persons	Level of control technologies utilized
Medical screening of nanotechnology workers	Autonomy Privacy Respect for persons	Appropriateness of the rationale for medical screening Extent to which participation is voluntary Maintenance of privacy test results
Investment in toxicological and control research	Nonmaleficence Justice Respect for persons	Adequacy of investment

**Table 2:** Summary of the state of knowledge for nanoparticle hazards and controls.

Awareness of knowledge	Content of knowledge (hazards and controls)
1. What we know we know	Health effects of ultrafines, air pollution, and fibers How to control ultrafine particles in the workplace Importance of size, surface area, and surface characteristics Serious health effects of some nanoparticles in animals Translocation of some nanomaterials along the olfactory nerve in animals
2. What we know we don’t know	Measurement and characterization techniques Hazards of newly engineered particles Extent of translocation in the body Interaction with contaminants in the workplace Importance of dermal exposure Health effects in workers Risks to workers Effectiveness of controls Advisability of medical screening and biological monitoring Risk to workers’ families
3. What we don’t know we know	Extensive experience available in controlling hazardous substances and agents (radiation, biological agents, pharmaceuticals) that can be applicable to nanoparticles Proprietary nanoparticle information Lessons from previous “new” technologies
4. What we don’t know we don’t know	Unanticipated new hazards Unanticipated new controls Wrong assumptions about hazards and controls
